# Ethnogeographic Distribution of Nigerian Fermented Foods and the Prospects for Bioeconomy Improvements

**DOI:** 10.3390/foods15173087

**Published:** 2026-08-31

**Authors:** Afolake A. Olanbiwoninu, Theresa A. Awotundun, Johnson F. Afolabi, Rachael O. Fashogbon, Omololu Fagunwa

**Affiliations:** 1 Department of Microbiology and Biotechnology, Ajayi Crowther University, P.O. Box 1066, Oyo 211271, Oyo State, Nigeria; aa.olanbiwoninu@acu.edu.ng (A.A.O.); jf.afolabi@acu.edu.ng (J.F.A.); ro.fashogbon@acu.edu.ng (R.O.F.); 2 Department of Microbiology, Olabisi Onabanjo University, P.O. Box 2002, Ago Iwoye 121001, Ogun State, Nigeria; 3 School of Biological Sciences, Institute for Global Food Security, Queen’s University Belfast, 19 Chlorine Gardens, Belfast BT9 5DL, UK

**Keywords:** Nigerian fermented foods, food security, circular bioeconomy, ethnogeographic distribution, microbial ecology, food safety, sustainable development

## Abstract

Fermented foods and beverages represent a cornerstone of Nigerian food culture, yet their scientific documentation remains fragmented, and their potential within global bioeconomy frameworks is largely unrealised. This review addresses two interrelated gaps: the absence of a comprehensive ethnogeographic analysis of Nigerian fermented foods that integrates ecological, cultural, and agricultural drivers of regional diversity, and the limited examination of these foods as models for circular bioeconomy development. Using a narrative synthesis of peer-reviewed literature spanning 1986–2025, we systematically map the distribution of fermented tuber, cereal, legume, dairy, and fruit products across Nigeria’s major geopolitical zones, demonstrating that agroecological endowment, ethnic composition, trade routes, and cross-cultural interactions shape regional variation. Beyond their socio-cultural significance, Nigerian fermented foods contribute meaningfully to food security by extending shelf life, enhancing nutrient bioavailability, and reducing post-harvest losses. This review further positions *ogi* (fermented maize gruel) and *garri* (fermented cassava granules) as model systems for circular bioeconomy integration, demonstrating how by-product valorisation can generate value-added outputs, including animal feed, bioethanol, biodegradable packaging, and organic acids. Key challenges, including food safety deficits, absence of standardised production protocols, and limited regulatory frameworks, are critically assessed. Three priority research directions are identified: metagenomics-based microbiome profiling, development of culturally appropriate starter cultures, and formulation of gender-responsive regulatory instruments. Nigerian fermented foods, properly documented and integrated into innovation systems, represent an underutilised asset for sustainable food system transformation in Sub-Saharan Africa.

## 1. Introduction

Fermentation is one of the oldest and most widespread food technologies in humanity, documented across virtually every culture and ecological zone [1]. In sub-Saharan Africa, fermented foods are dietary staples, vehicles for cultural identity, mechanisms of post-harvest preservation, and subjects of biotechnological interest for their probiotic potential [2], antimicrobial activity, and functional food properties [3,4]. Nigeria, as the most populous nation on the continent and one of its most ecologically and ethnically diverse, hosts a particularly rich repertoire of fermented food traditions, spanning tuber-based products such as *garri* and *fufu*, cereal ferments such as *ogi* and *burukutu*, legume condiments such as *iru* and *ugba*, and dairy products such as *nono* [5,6].

Despite their prevalence, Nigerian fermented foods remain substantially understudied relative to their scientific and economic significance. Prior reviews have catalogued individual products or focused on specific microbial communities, but few have adopted an explicitly ethnogeographic lens that examines how ecological gradients, ethnic composition, agricultural resource availability, and historical trade patterns collectively shape the spatial distribution of fermentation traditions [7,8]. Without understanding why particular foods are produced where they are, opportunities for targeted innovation, commercialisation, and policy support will remain suboptimal.

A second significant gap concerns the relationship between traditional fermented foods and the circular bioeconomy. As global food systems face mounting pressure from climate change, population growth, and resource scarcity, there is growing interest in production models that valorise by-products and operate through closed material loops [9]. High-volume staples such as *garri* and *ogi* generate substantial processing residues whose valorisation potential remains largely unexplored in the scientific literature.

This present review differs in bringing these two strands together under a single integrated lens, treating ecological, ethnic, and historical drivers as jointly explanatory of regional distribution, and treating that same regional logic as the analytical basis for the bioeconomy assessment that follows, rather than treating ethnogeography and bioeconomy potential as separate bodies of literature.

### Scope and Methodology

This review employed a narrative synthesis methodology, drawing on peer-reviewed articles, book chapters, and authoritative reports identified through searches of Web of Science, Scopus, Google Scholar, and PubMed, using the terms: ‘Nigerian fermented foods’, ‘traditional fermentation Africa’, ‘cassava fermentation’, ‘ogi’, ‘garri bioeconomy’, and related variants. Sources were selected for relevance, methodological quality, and temporal coverage (1986–2025), with priority given to primary research and systematic reviews in peer-reviewed journals. Where evidence was conflicting or limited, this is explicitly noted. The geographic scope encompasses the principal ecological zones in Nigeria treated as culturally and agroecologically meaningful units of analysis.

## 2. Historical Background and Classification of Nigerian Fermented Foods

### 2.1. Historical and Pre-Colonial Roots

Fermentation as a food preservation strategy predates colonial contact in Nigeria by centuries. Indigenous kingdoms and ethnic communities developed sophisticated fermentation practices to preserve seasonal harvests, ensure a year-round food supply, detoxify antinutritional compounds, and create culturally distinctive flavours. Cassava fermentation served the dual purpose of preserving the highly perishable tuber and reducing its cyanogenic glycoside content, a critical food safety function [10]. The alkaline fermentation of African locust beans (producing ‘daddawa’ or ‘iru’) enabled preservation of a high-protein condiment capable of surviving the West African wet season [6].

European contact from the fifteenth century introduced new crops, notably maize, that were rapidly integrated into existing fermentation traditions, generating products such as maize *ogi* alongside older sorghum and millet variants [5]. Core fermentation practices persisted through indigenous knowledge systems transmitted across generations, particularly among women who have historically been the primary practitioners of household-scale fermentation [11].

### 2.2. Classification of Nigerian Fermented Foods

Nigerian fermented foods are systematically classified by substrate type, which broadly corresponds to agroecological zones and ethnic traditions. Five principal categories are recognised: (1) tuber-based products, primarily from cassava (‘garri’, ‘fufu’, ‘lafun’, ‘abacha’); (2) cereal-based products from maize, sorghum, and millet (‘ogi’, ‘kunun-zaki’, ‘burukutu’, ‘pito’, ‘masa’); (3) legume-based condiments (‘iru/daddawa’, ‘ugba’, ‘ogiri’, ‘afiyo/okpehe’); (4) dairy products (‘nono’, ‘fura da nono’); and (5) fruit and palm-sap beverages (‘agadagidi’, ‘emu’/palm wine). The comprehensive summary of some popular fermented foods, the associated fermentative microbes and the base substrates is provided in Table 1.

## 3. Ethnogeographic Analysis: Regional Drivers of Fermentation Diversity

**Figure 1:**
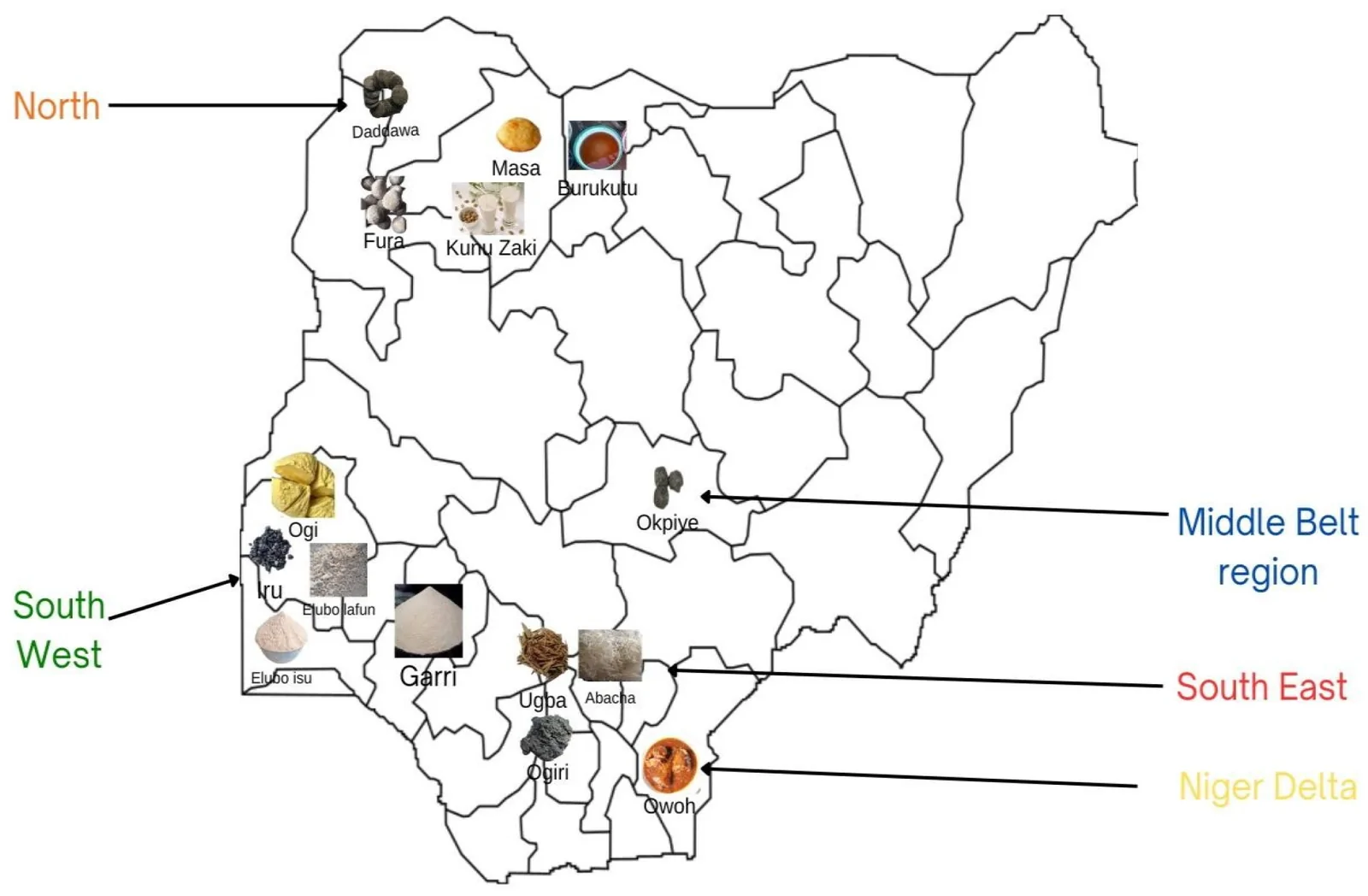
Unique fermented food specialties in different regions of Nigeria. Sources: North region: [53,54,55]; Southwest: [6,19,28,47,56,57,58]; Middle-belt region: [56]; Southeast region: [16,56,59]; Niger-Delta region: [56].

Ethnogeography, as applied to food systems, examines how ecological endowment, ethnic identity, historical processes, and agricultural resource availability interact to shape food traditions across space. In Nigeria, regional fermentation diversity reflects a coherent logic; communities ferment what they grow in ways shaped by cultural identity and adapted to local climate and resource constraints. The geographic distribution of distinctive fermented food specialties across Nigeria’s principal ecological and cultural zones is illustrated in Figure 1. It reveals an asymmetry in how thoroughly each zone’s fermentation repertoire has been documented. The North and Southwest boxes are populated with *daddawa*, *fura*, *masa*, *kunun-zaki*, and *burukutu* in the North; *ogi*, *iru*, *elubo*/*isu*, and *garri* in the Southwest; while the Southeast and Niger Delta are represented by *ugba* and *abacha*; *ogiri* and *owoh*; and the Middle Belt is represented by *okpiye*. It is also worth noting that the map’s single-region assignment simplifies products that are, in practice, consumed well beyond their labelled zone; *garri* and *daddawa* in particular are eaten nationally, so the figure is best read as indicating where each product is most strongly associated and most extensively documented, rather than where it is exclusively produced or consumed.

### 3.1. North and Sahel Zone: Arid Adaptations and Pastoral Dairy Traditions

The northern states, dominated by Hausa and Fulani populations, occupy a semi-arid Sahel ecology characterised by low rainfall and agricultural systems centred on sorghum, millet, and cattle pastoralism. These agroecological conditions have directly shaped the predominance of grain-based and dairy-based fermented foods. *Masa*, *kunun-zaki*, *burukutu*, *daddawa*, *nono*, and *fura da nono* are staple products whose production reflects available grain resources and the preservation requirements of a hot, dry climate.

*Masa*, a fried rice- or sorghum-based fermented cake, and *kunun*-*zaki*, a spiced non-alcoholic millet/sorghum beverage, are consumed daily across the northern states as breakfast and street-vended foods, and both depend on spontaneous lactic and yeast fermentation of the same grain base that underlies *burukutu.* Masa production typically combines rice or sorghum flour with a portion of previously fermented batter as backslopping inoculum, a practice that parallels sourdough-type propagation and that has received little systematic microbiological study. The prevalence of *nono* directly reflects the Fulani pastoral tradition; the ready availability of milk from cattle herds made dairy fermentation a practical and nutritionally important food strategy [53]. Knowledge of dairy fermentation is transmitted almost exclusively through Fulani women, often termed ‘milkmaids’, who traditionally learn nono and fura da nono production from mothers or senior female relatives and control its trade, including door-to-door and market hawking [60].

### 3.2. Middle Belt: Ethnic Plurality and Fermentation Diversity

The Middle Belt, occupying the transition zone between the Sahel and humid south, is characterised by remarkable ethnic plurality and a correspondingly diverse culinary landscape. The Igala and Idoma peoples of Benue State produce *okpiye*/*afiyo*, an alkaline-fermented condiment from African mesquite (*Prosopis africana*) seeds [61].

Beyond *okpiye*/*afiyo*, the Middle Belt’s fermentation repertoire includes products less documented outside regional food-science literature. Fermented yam and cassava derivatives such as *ibe* (yam flour; *omu ibe* in Igala, *Ibe* in Idoma, equivalent to *amala* in Yoruba) and *ikwurikwu* are processed alongside more widely known products like *garri* and *fufu*, reflecting the zone’s shared cassava-growing ecology with the south. *Ikwurikwu* is a fermented cassava product, moulded into the shape of a ball, and made into dough in boiling water before consumption with any desired soup. The cassava used is the bitter variety [62]. This product diversity is a direct expression of the exceptional ethnic plurality in the Middle Belt; the region is home to more than twenty distinct ethnolinguistic groups, including the Tiv, Nupe, Igala, Idoma, Berom, and Eggon, each contributing locally distinct processing methods, naming conventions, and product variants to a shared repertoire of substrates. This ethnic fragmentation, combined with the transitional agroecology between the Sahel and the humid south, produces a fermentation landscape that resists the more unified regional identity seen in the Southwest or Southeast, and correspondingly has received less systematic scientific documentation.

The transitional ecology of the Middle Belt enables a broader fermentation repertoire than that of the arid north, while its historically diverse ethnic composition has generated significant variation in processing methods and culinary applications of shared raw materials.

### 3.3. Niger Delta and Mid-West: Aquatic Resources and Distinctive Condiments

The distinctive aquatic ecology and cultural traditions of the Niger Delta have generated unique fermented products not found elsewhere in Nigeria. *Owoh*, a condiment produced by the Urhobo and Itsekiri peoples from fermented cotton seeds (*Gossypium hirsutum*) or African yam beans (*Sphenostylis stenocarpa*), exemplifies how the availability of local resources shapes fermentation traditions [63]. The riverine economy of the region supports the consumption of *burukutu* and locally distilled *ogogoro* (Raffia palm gin), embedded in the social and ceremonial life of riparian communities [57].

*Ogogoro* (also known as sapele water, *kai-kai*, or push-me-I-push-you) represents a further fermentation tradition specific to the Niger Delta’s raffia-palm-dominated wetland ecology. Originating as a local response to import restrictions on foreign spirits during the 1930s, *ogogoro* production remains an important informal-sector livelihood in riverine Niger Delta communities, though it continues to operate largely outside formal regulatory oversight, with implications for both product safety and the broader food-safety governance challenges [64].

### 3.4. Cassava Centrality and Igbo Fermentation Heritage

The southeastern states, predominantly Igbo, are characterised by a humid rainforest ecology with high cassava productivity. The centrality of cassava to Igbo food culture is reflected in the diversity of fermented products; *garri* (in the palm-oil-enriched yellow form characteristic of the southeast), *fufu* (*akpu*), and *abacha* are emblematic Igbo foods [16,59]. The fermented legume condiments *ogiri* and *ugba*/*ukpaka* serve as important sources of protein and flavour and are deeply embedded in Igbo culinary and ceremonial traditions [5,56]. *Ugba*/*ukpaka* in particular is strongly associated with festive and ceremonial consumption in Igbo communities, prepared in larger quantities for gatherings and celebrations rather than only everyday use. Most notably, the New Yam Festival (*Iwa Ji*/*Iri Ji Ohuru*), an annual harvest celebration in which food plays a central ritual role, yams and accompanying dishes are ceremonially offered before communal feasting begins.

### 3.5. Southwest: Yoruba Fermentation Traditions and Botanical Diversity

The southwestern states, home to the Yoruba, occupy a sub-humid ecological zone that supports a wide range of food crops and a highly diverse fermentation repertoire. *Iru* (alkaline-fermented African locust bean, *Parkia biglobosa*) is the southwestern counterpart of northern *daddawa*. However, in the southwest, an important regional variation is that a softening agent called *kuru* (containing sunflower seeds and *trona*/*kaun*, sodium sesquicarbonate) is incorporated into the pulp during processing in some areas, resulting in *iru-pete* [65]. *Ogi* serves as infant weaning food, convalescent diet, ceremonial offering, and fasting food, embedding it deeply in Yoruba social practice beyond its nutritional role [19]. It is usually eaten with *akara* (fried bean balls) by children and adults as a popular breakfast across the region. *Elubo-lafun* and *Elubo-isu* are fermented cassava and yams, respectively, in fibrous, powdered form. Their flours are usually added to boiling water with constant stirring until a black/brownish, smooth, thick paste (*amala*) forms. The paste is served with a variety of soups, such as efo riro, okro, or ewedu, and *gbegiri* [6]. *Amala*, with the indigenous soups “ewedu and gbegiri,” is usually one of the important dishes in Yoruba events. Some alcoholic beverages, such as *Pito* [28], *Agadagidi* [47,66], *Ogogoro* [57] and *Otika* [58], are native to the Yoruba people.

### 3.6. Cross-Cultural Influences and Food Exchange

*Garri* and *fufu* are consumed across both southwest and southeast Nigeria, with notable regional variation that reflects cross-cultural exchange. Southeastern Igbo communities typically enrich *garri* with palm oil during frying (yielding ‘yellow garri’), while southwestern Yoruba prefer uncoloured white *garri*. These variations reflect access to palm oil, cultural aesthetic preferences, and historical trade patterns [67,68]. Interstate migration and urban food markets have progressively spread foods beyond their regions of origin, raising quality-control challenges as production moves beyond the context of traditional knowledge and community oversight.

## 4. Production Methods, Microbiology, and Nutritional Attributes

The regional patterns described above are not merely descriptive geography: the substrate available in a given zone dictates the dominant fermentation type (lactic acid bacteria-driven acidification for grain and tuber ferments versus alkaline, *Bacillus-*driven fermentation for protein-rich legume condiments such as *iru* and *ogiri*), which in turn shapes the microbial communities that establish themselves and the nutritional transformations that result. The cassava-centred fermentation of the southeast and southwest, for instance, is dominated by lactic acid bacteria and cyanogenic-glycoside-detoxifying yeasts, while the sorghum- and locust-bean-based fermentations of the north and Middle Belt favour *Bacillus* species and different *Lactobacilli*.

### 4.1. Tuber-Based Fermented Products

Cassava (*Manihot esculenta*) fermentation serves the critical dual function of producing distinctive food products and reducing cyanogenic glycoside content (linamarin and lotaustralin). Enzymatic hydrolysis of these compounds by beta-glucosidases, facilitated by microbial activity, releases hydrogen cyanide that volatilises before consumption [10]. The degree of detoxification, however, is variable and depends on fermentation duration, temperature, and microbial community composition, a food safety consideration of ongoing relevance.

#### 4.1.1. Garri

**Figure 2:**
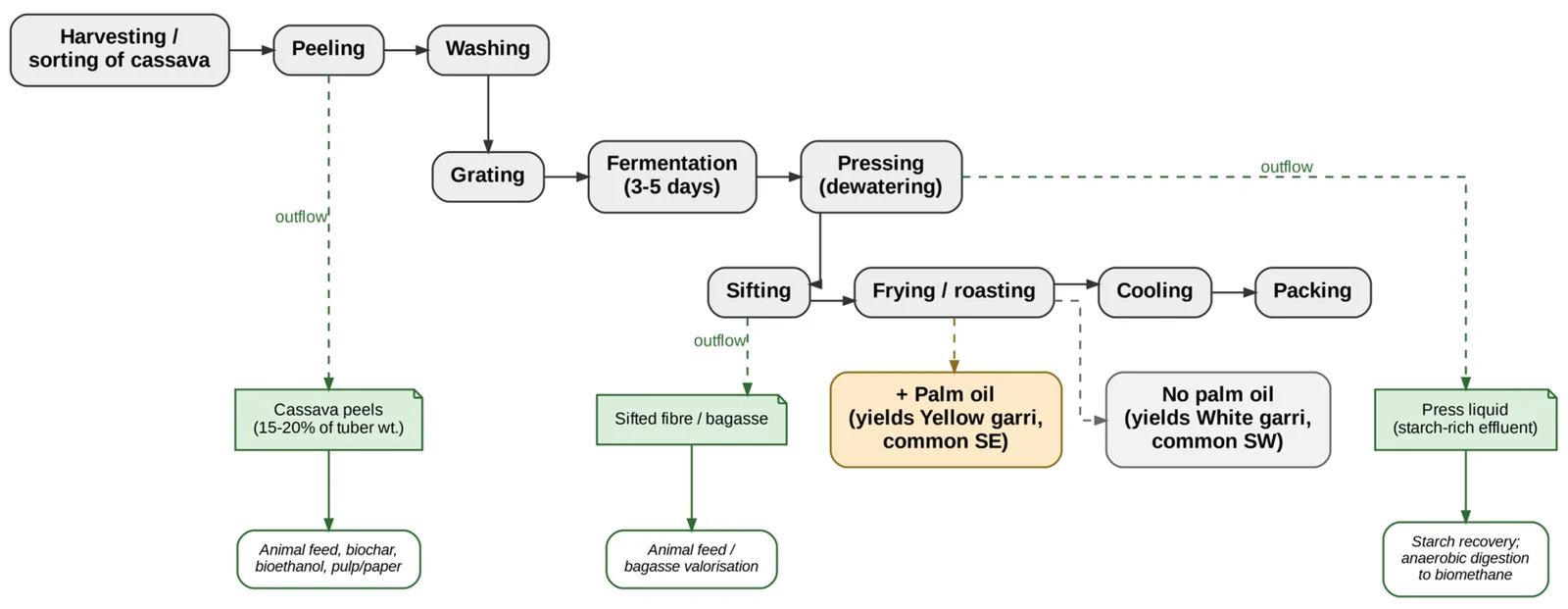
Process flow chart for “garri” production. Solid black arrows indicate the main sequence of processing steps. Dashed green arrows indicate material outflows generated at each stage.

*Garri*, a coarse granulated partially gelatinised cassava product, is produced by peeling, washing, grating, pressing, fermenting (3–5 days), and roasting fresh cassava [69,70,71]. The production process is shown in Figure 2. The fermentation is primarily driven by lactic acid bacteria (LAB), principally *Lactiplantibacillus plantarum* and *Leuconostoc* species and yeasts, including *Saccharomyces cerevisiae* and *Kluyveromyces marxianus* [16]. Acidification by LAB and yeast establishes rapidly over the 3–5 day fermentation, driving the pH decline that both develops the product’s characteristic sourness and suppresses spoilage and pathogenic organisms, a key contributor to garri’s long shelf life relative to unfermented cassava products. The resulting product has a characteristic sour taste and low moisture content, conferring extended shelf life. This product is a staple ingredient in preparing *eba* (paste eaten with soup). Nutritionally, *garri* is primarily a carbohydrate source; its protein and micronutrient content are limited, a public health consideration given its role as a dietary staple for lower-income populations [72]. Hence, *garri* is often paired with complementary foods like beans, coconut, dried fish, groundnut, *kulikuli*, and *donkwa* (groundnut products) when soaked in cold water, thereby enriching the diet with a balanced nutritional profile. In numerous homes, *Garri* is a preferred lunch option, contributing significantly to the cuisine [72].

#### 4.1.2. Fufu and Lafun

**Figure 3:**
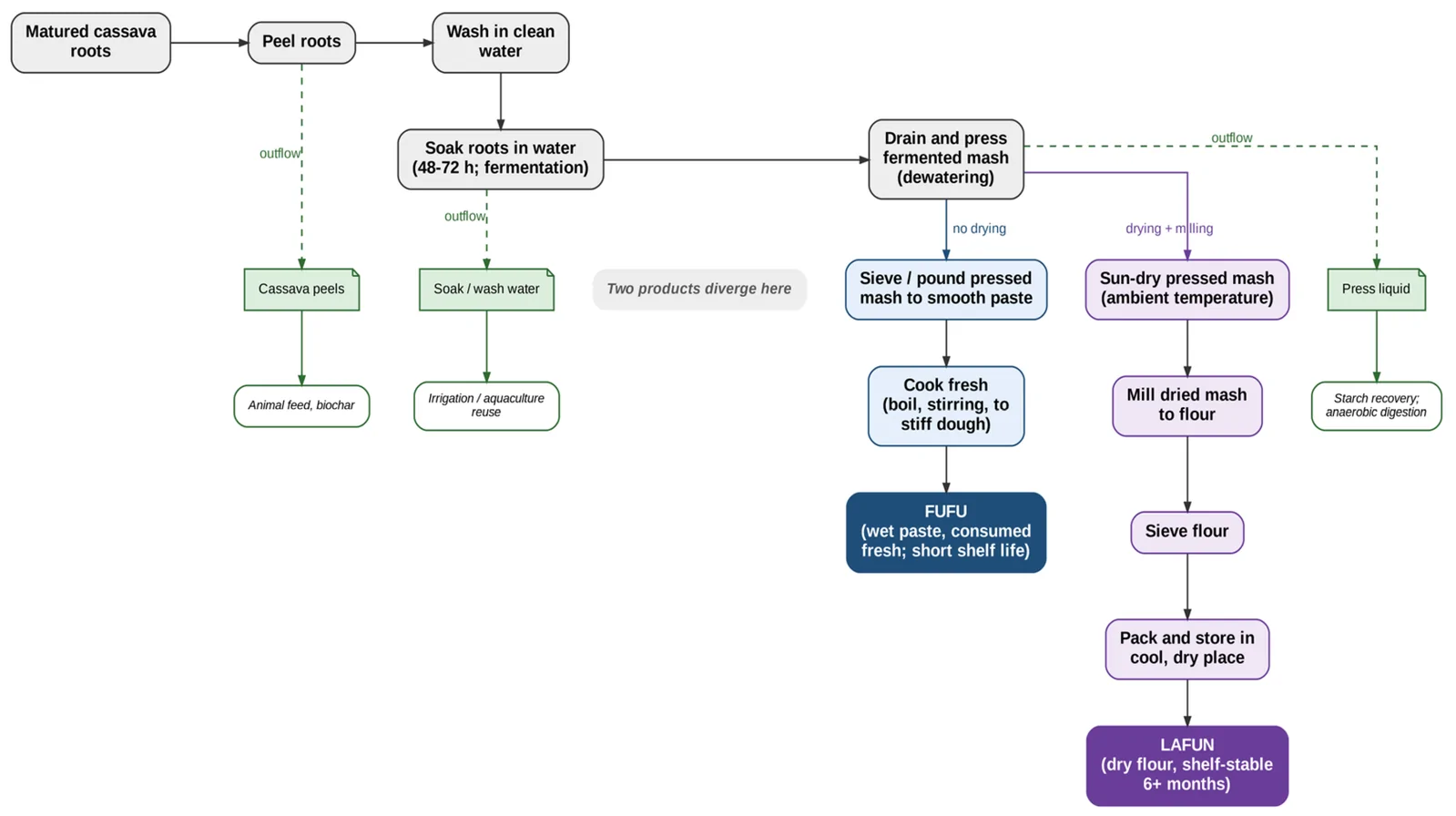
Combined process flowchart for “fufu” and “lafun” production showing the shared initial processing stages and the point at which the two products diverge. Solid black arrows indicate the main sequence of processing steps. Dashed green arrows indicate material outflows generated at each stage.

**Figure 4:**
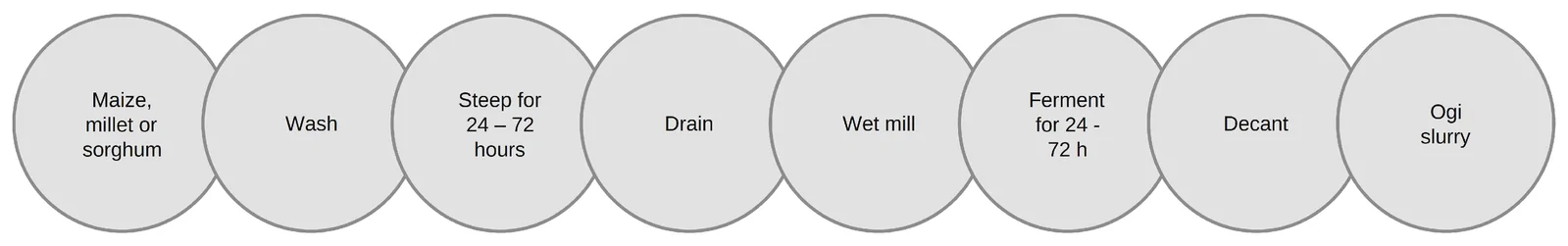
Process flowchart for *Ogi* production.

*Fufu* is made by submerging peeled cassava roots in water for 2–3 days, then pounding and cooking (Figure 3). The fermentation involving the broad genera (*Lactiplantibacillus*, *Leuconostoc*) reduces pH, softens tissue, and partially reduces cyanogen [10]. *Lafun* undergoes a similar initial fermentation, but the mash is sun-dried and ground into a fibrous flour (Figure 4), with a shelf life of 6 months or more under appropriate storage conditions [73].

#### 4.1.3. Abacha

*Abacha*, also known as African salad, is prepared by fermenting peeled, washed, and shredded cassava, after which supplementary ingredients such as vegetables, oil, fish, pepper, and seasoning can be added. It can be eaten at any time of day as a snack or a main meal [16,59].

### 4.2. Cereal-Based Fermented Products

Cereal-based fermented foods constitute a substantial proportion of staple diets across Nigeria, serving as weaning foods for infants, functional foods for children, and important sources of energy and micronutrients for the general population [3,74]. The principal substrates are sorghum (*Sorghum bicolor*), millet (*Panicum miliaceum*), maize (*Zea mays*), and rice (*Oryza sativa*). During fermentation, these grains undergo biochemical and nutritional modifications, predominantly driven by lactic acid bacteria (LAB), which produce organic acids, bacteriocins, and volatile compounds that enhance food safety, shelf life, and organoleptic properties [75]. Products range from fermented slurries and porridges, such as ogi, to alcoholic beverages, such as *burukutu* and *pito*, and non-alcoholic drinks, such as *kunun-zaki*.

#### 4.2.1. Ogi

*Ogi* is a fermented cereal slurry produced primarily from maize, though sorghum and millet variants are common. Maize grains are soaked for one to two days, wet-milled, sieved, and allowed to ferment and sediment before being sold as wet cakes wrapped in leaves. The fermentation involves a microbial succession in which *Corynebacterium* spp. emerge during the early steeping phase, followed by the establishment of LAB, principally *Lactiplantibacillus plantarum* and yeasts including *Saccharomyces cerevisiae,* during which *the* acidification process occurs, suppressing spoilage/pathogenic organisms [76]. This succession is functionally significant beyond acidification; LAB metabolism during the sedimentation phase measurably alters the amino acid and vitamin profile of the slurry, relevant to its role as a primary infant weaning food where nutrient density is a public health concern. The flowchart for *ogi* processing is presented in Figure 4. Most published microbiological characterisations of *ogi* relied on culture-dependent methods; metagenomics studies of analogous West African ferments suggest the true microbial complexity is substantially greater [32]. *Ogi* occupies a central socio-cultural position among the Yoruba and other southern Nigerian ethnic groups, functioning as the canonical infant weaning food, a convalescent diet, a ceremonial offering, and a fasting food [19,77]. *Koko*, a closely related product with lower viscosity, is made from maize and is typically consumed hot as a morning or evening beverage.

#### 4.2.2. Masa

*Masa* (*Waina*) is a fermented puff batter produced from rice, millet, maize, or sorghum, cooked in a characteristic cup-shaped pan. Widely consumed across the northern states by all ethnic groups, *masa* serves as both a staple snack and a significant source of income for women producers [6]. Masa fermentation is driven by *Lactiplantibacillus plantarum*, *Pediococcus acidilactici*, and *Limosilactobacillus fermentum*, alongside the yeast *Saccharomyces cerevisiae*, with the pH falling from approximately 6.0 to below 4.0 as bacterial activity dominates the process [6]. Despite its broad popularity, *masa* has received comparatively little scientific attention compared with *ogi*, highlighting a gap in the characterisation of northern Nigerian cereal ferments.

#### 4.2.3. Burukutu and Pito

*Burukutu* and *pito* are alcoholic beverages produced by malting and multi-stage fermentation of sorghum, millet, or maize. *Burukutu*, favoured in northern Nigeria, is characterised by a vinegar-like flavour and a cloudy brown appearance. Its fermentation involves a sequential microbial process: during the first 24 h, pH drops from approximately 6.2 to 4.3, declining further to 3.7 by 48 h, with *Acetobacter* and *Candida* species dominating the mature product; a pre-maturation boiling step eliminates residual LAB and yeasts [6,78]. This controlled elimination step is a rare example of an indigenous process that explicitly manages microbial succession to ensure product consistency, distinguishing *burukutu* from most other spontaneously fermented Nigerian products. *Pito*, originating among the Benin people of the Mid-West but consumed nationally and in neighbouring Ghana, is produced by a similar malting and fermentation process with *Geotrichum candidum*, *Lactobacillus* species, and *Candida* species as key fermentative organisms, yielding a dark brown liquid with approximately 3% alcohol by volume [28,79].

#### 4.2.4. Kunun-Zaki

*Kunun-zaki* is a popular non-alcoholic millet-based beverage originating in northern Nigeria and increasingly consumed in the south. Millet grains are steeped, wet-ground with spices (pepper, ginger, cloves), sieved, partially gelatinised, sweetened, and bottled. A brief LAB and yeast fermentation occur during the steeping phase (8–48 h). The product is shelf-stable at room temperature for approximately 24 h, a limitation that constrains commercial distribution; pasteurisation at 60 °C for 1 h, followed by refrigeration, extends shelf life to approximately 8 days [80], though adoption of such post-processing steps remains limited among traditional producers.

### 4.3. Legume-Based Fermented Condiments

Fermented legume condiments represent a nutritionally and culturally significant category of Nigerian fermented foods, distinguished from cereal and tuber ferments by their alkaline fermentation chemistry and high protein content. Unlike the acid fermentations driven by LAB that characterise cereal and cassava products, legume fermentation in Nigeria is dominated by members of the genus *Bacillus*, principally *B. subtilis*, *B. circulans*, *B. licheniformis*, and *B. coagulans*, whose proteolytic and lipolytic activity solubilises and partially hydrolyses seed proteins, substantially improving their digestibility and bioavailability [3,81]. The alkaline environment generated also reduces antinutritional factors, including trypsin inhibitors and tannins, making these condiments an important traditional strategy for combating protein-energy malnutrition in communities with limited access to animal-source foods [82,83]. Despite their nutritional importance, these condiments have not been successfully commercialised at scale, owing to short shelf lives, inconsistent quality resulting from uncontrolled spontaneous fermentation, and difficulties meeting packaging specifications for wider markets [84,85].

#### 4.3.1. Iru and Daddawa

**Figure 5:**
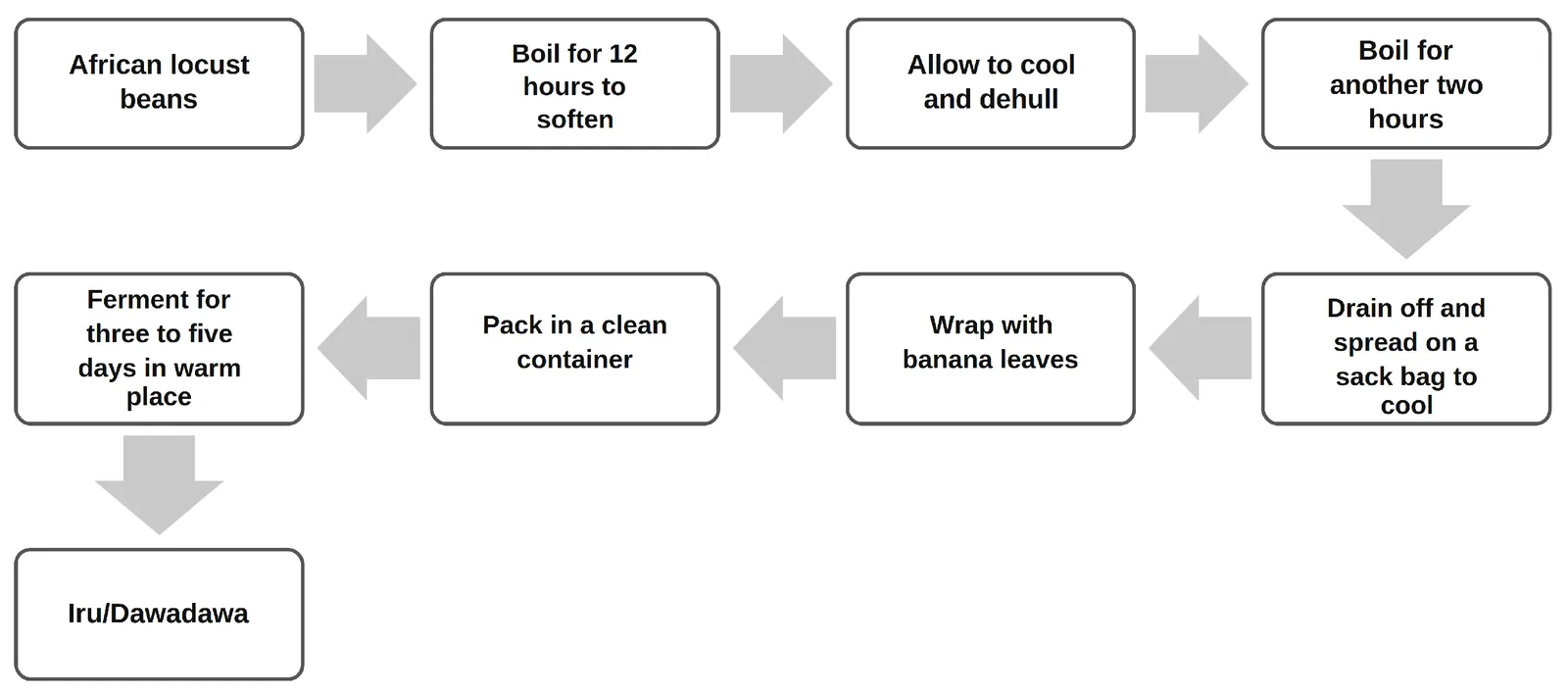
Process flowchart for “iru”/ “dawadawa” production.

*Iru* (southwestern Nigeria) and *daddawa* (northern Nigeria) are regional names for the same class of condiment produced from the alkaline solid-substrate fermentation of African locust bean (*Parkia biglobosa*) seeds. The seeds are boiled for approximately 12 h to soften the testa and facilitate hand dehulling, then boiled again for 2 h to further soften the cotyledons, wrapped in banana leaves, packed in a closed container, and left to ferment at room temperature [6] (Figure 5). The resulting product is rich in fat (39–40%) and protein (31–40%) and contributes substantially to energy intake, protein, and riboflavin across many communities in West and Central Africa [86]. It functions as a flavour enhancer and low-cost meat substitute in soups and stews, serving a culinary role broadly comparable to that of bouillon cubes. Despite identifying the principal fermentative organisms, this knowledge has not been translated into controlled industrial production; the condiment remains entirely artisanal, with quality variability that limits its market potential.

#### 4.3.2. Ugba

*Ugba* (*ukpaka*), produced from the African oil bean (*Pentaclethra macrophylla* Benth), is a cherished staple of the eastern Igbo region of Nigeria. Seeds are boiled for three hours, dehulled, sliced into thin pieces (0.5–1 mm), boiled again for two hours, then repeatedly rinsed, steeped in cold water for four hours to remove residual bitterness, wrapped in banana leaves (*Musa sapientum*), and allowed to ferment at room temperature [87]. The resulting product contains approximately 44% protein by dry weight and contains at least 17 essential amino acids [88].

#### 4.3.3. Ogiri

*Ogiri* is a pungent, oily paste produced from the fermentation of castor oil seeds (*Ricinus communis*), melon seeds (*Citrullus vulgaris*), or fluted pumpkin seeds (*Telfairia occidentalis*), depending on seasonal availability and regional preference. Seeds are boiled for 2 h until visibly softened and colour changed, dehulled, rinsed, boiled for a further hour, cooled, wrapped in banana leaves, and fermented at room temperature.

#### 4.3.4. Afiyo/Okpehe

*Afiyo* (*okpehe*), produced from *Prosopis africana* seeds, like *iru* and *ugba*, undergoes moist, solid-state alkaline fermentation by chance inoculation, yielding a sticky, browned seed mash with a concentrated, pungent aroma that serves as a versatile flavouring agent [61].

### 4.4. Dairy and Fruit-Based Products

The principal production technique is backslopping: the addition of a small quantity of previously fermented milk, as an undefined starter culture, to freshly extracted cow milk, a method that maintains microbial continuity across fermentation batches while simultaneously perpetuating variability in product quality and carrying inherent food safety risks [89].

#### Nono

**Figure 6:**
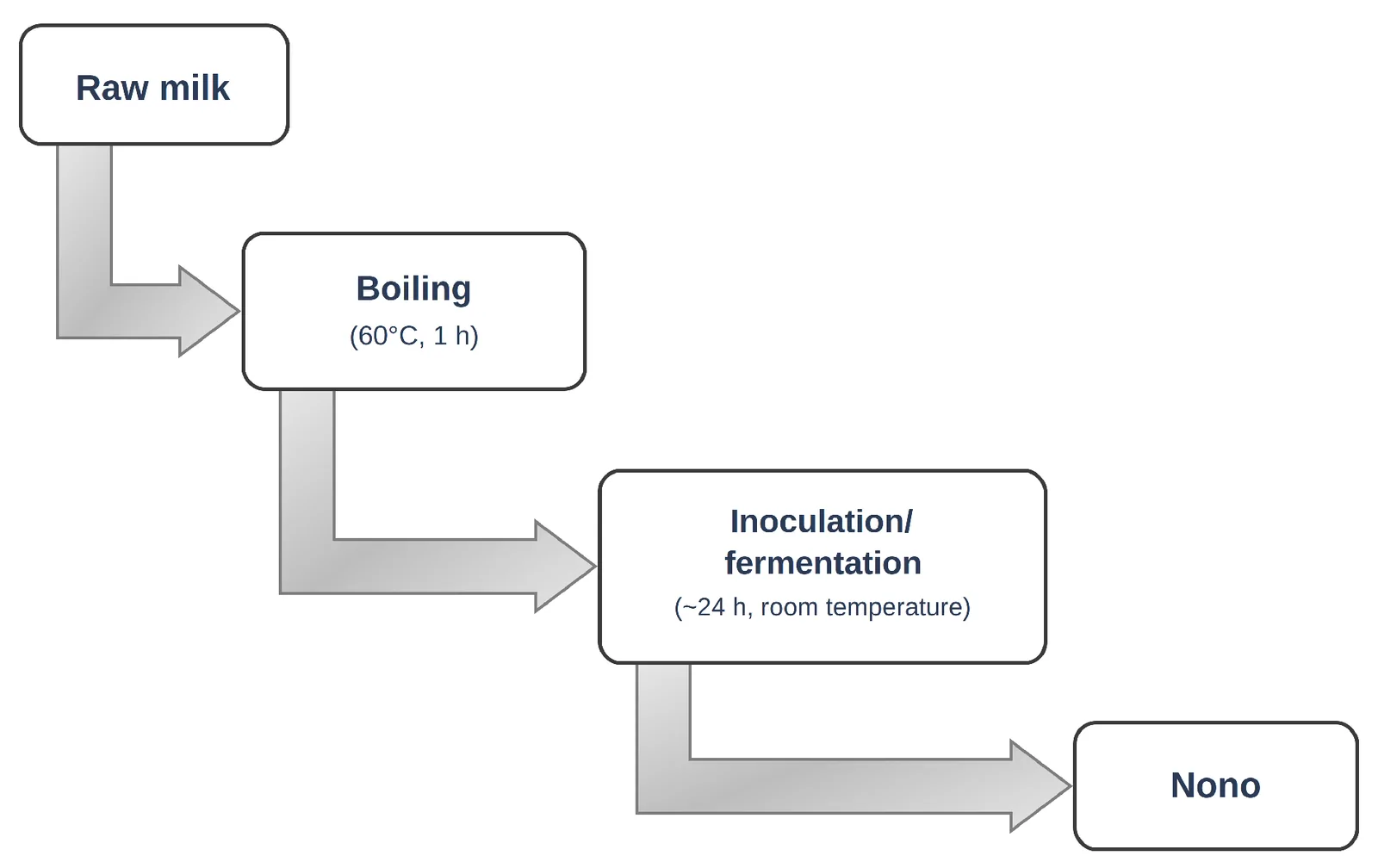
Process flow chart for the production of *nono*.

*Nono*, the most widely consumed Nigerian fermented dairy product, is produced from cow milk collected between the third and sixth months of lactation. Fresh milk is inoculated with previously fermented milk and allowed to ferment at room temperature for approximately 24 h, during which the resident microbial community converts lactose to lactic acid, yielding the characteristic sharp, yoghurt-like taste of the finished product (Figure 6. The microbial ecology of *nono* is considerably more complex than early culture-dependent studies suggested. Molecular characterisations have identified a diverse community comprising *Lactiplantibacillus plantarum* and *Lactobacillus delbrueckii* subsp. *bulgaricus*, *Lactococcus lactis* subsp. *Lactis* and *cremoris*, *Limosilactobacillus fermentum*, *Ligilactobacillus salivarius*, *Streptococcus infantarius* subsp. *infantarius*, and yeasts including *Saccharomyces cerevisiae*, *Rhodotorula mucilaginosa*, *Trichosporon* species, and *Geotrichum penicillatum* [55,89,90]. The presence of *Staphylococcus* species and enterococci in some characterisations warrants ongoing public health attention, particularly given consumption of *nono* across all age groups without heat treatment before serving. Recent metagenomics studies of analogous West African grain ferments have revealed substantially greater complexity [23,32], with significant implications for quality control and starter culture development.

Nutritionally, *nono* is a valuable source of protein, essential amino acids, calcium, phosphorus, and vitamins A, B complex, C, and E, though iron and ascorbic acid levels are low, consistent with other milk-based fermented products [91,92]. It is commonly consumed in combination with *fura*: millet or sorghum flour compressed into spiced balls and briefly boiled. The pairing is known as *fura da nono*, which increases the protein and energy density of the meal [6].

### 4.5. Fermented Fruit and Palm-Sap Products

Fruit and palm sap fermentation, while less extensively documented than cereal, tuber, and legume fermentations in the Nigerian literature, constitutes a meaningful component of the fermented food repertoire.

#### 4.5.1. Agadagidi

*Agadagidi* is a traditional alcoholic beverage produced from overripe bananas or plantains (*Musa* spp.), characterised by a cloudy, effervescent appearance and a sweet-sour taste profile typical of African traditional alcoholic beverages [93]. The fermentation involves a mixed microbial community including *Bacillus subtilis*, *Lactiplantibacillus plantarum*, *Leuconostoc mesenteroides*, *Saccharomyces cerevisiae*, and *Candida utilis* [46].

#### 4.5.2. Palm Wine

*Emu* (palm wine), produced from the sap of the oil palm (*Elaeis guineensis*) or Raphia palm (*Raphia hookeri*), represents the most widely consumed fermented fruit-sap beverage in Nigeria. Freshly tapped palm sap undergoes rapid spontaneous fermentation driven by yeasts, principally *Saccharomyces cerevisiae* and *Hanseniaspora uvarum*, alongside LAB including *Lactiplantibacillus plantarum* and *Leuconostoc mesenteroides*, resulting in an initially sweet, progressively souring and alcoholising beverage whose composition changes substantially within hours of tapping [48,52]. The highly perishable nature of palm wine, which begins to deteriorate organoleptically within 24 h at ambient temperature, represents a significant constraint on its commercialisation and underlies the limited scientific attention it has received relative to its cultural and economic importance across Nigeria.

## 5. Nigerian Fermented Foods and Global Food Security

Fermented foods represent a low-cost, low-technology, and culturally embedded strategy for improving food availability, safety, and nutritional quality [94]. The relevance of Nigerian fermented foods to food security operates through three interconnected pathways.

### 5.1. Preservation and Post-Harvest Loss Reduction

Fermentation extends the shelf life of highly perishable tropical substrates, such as fresh cassava, milk, and locust beans, by creating acidic or alcoholic environments that are hostile to spoilage organisms. This is of particular importance in rural Nigeria, where refrigeration infrastructure is limited and post-harvest losses of fresh cassava can reach 40% of production within 24–48 h of harvest [95]. Converting fresh cassava into *garri*, which has a shelf life of months without refrigeration, effectively eliminates a large fraction of this post-harvest loss, with direct implications for household food security.

### 5.2. Nutritional Enhancement

Fermentation improves nutritional quality through several mechanisms: degradation of antinutritional factors (phytates, tannins, oxalates) that inhibit mineral bioavailability; partial hydrolysis of complex carbohydrates and proteins improving digestibility; reduction of cyanogenic glycosides in cassava; and, in some products, synthesis of B vitamins and short-chain fatty acids by microbial metabolism [96]. These effects are nutritionally meaningful in populations whose diets are dominated by a small number of staple crops. The weaning role of *ogi* is particularly significant, though the energy density and protein content of *ogi* are modest, and supplementation or enrichment strategies are recommended for exclusive *ogi* feeding [97].

### 5.3. Food Sovereignty, Gender, and Resilience

Traditional fermented food production supports food sovereignty by enabling communities to transform locally available agricultural resources into diverse and stable food products without dependence on external inputs or cold-chain infrastructure [98]. This self-reliance confers resilience against climate shocks that disrupt growing seasons or supply chains, and supports rural livelihoods, particularly for women [11,99].

The gender dimension of fermented food production deserves explicit recognition. Across all five regions analysed, women are the primary producers, traders, and custodians of knowledge related to traditional fermentation. They are estimated to account for approximately 80% of food processing activity in Nigeria overall, and value-chain mapping studies of *garri* processing specifically find that women constitute the near-totality of labour at the peeling, washing, fermenting, and packaging stages, while men are more often concentrated in the earlier stages of cultivation and bulk marketing [100]. Commercialisation strategies that fail to account for this risk marginalise women from economic benefits or disrupt the knowledge systems upon which fermentation quality depends. Future policy and investment frameworks must incorporate gender-transformative approaches to the development of fermented foods.

## 6. Fermented Foods as Circular Bioeconomy Models

The circular bioeconomy represents a model of production that eliminates waste by designing material flows as closed loops, with by-products of one process serving as inputs to another [9]. Traditional fermented food systems, with their reliance on biological transformation of agricultural residues and generation of diverse by-product streams, are natural candidates for bioeconomy integration.

### 6.1. Ogi: Circular Bioeconomy Integration

Approximately one quarter of Nigeria’s population is estimated to consume *ogi* on a weekly basis, reflecting its dual role as an infant weaning food and adult breakfast staple [101]. *Ogi* production remains largely small-scale and household- or micro-enterprise-based; proposals to industrialise the process face constraints around cost, quality control, and infrastructure to those limiting formalisation [102]. *Ogi* production generates three principal by-product streams: cereal bran from wet-milling and sieving, steep water from grain soaking, and spent water from milling. All three by-products carry significant valorisation potential.

#### 6.1.1. Utilisation of Cereal Bran and By-Products

The cereal bran from the milling process is rich in dietary fibre, proteins, and micronutrients [103]. Thus, this bran can be processed into fibre-rich supplements or fortified food products and returned to the food chain for better nutrition. Ogunmuyiwa et al. [104] used corn bran to fortify extruded snacks made with starch and *bambara* nut flour. This increased the total dietary fibre content of the extrudates to above 7%. Likewise, de Erive et al. [105] and Pauline et al. [106] enriched bread with corn bran to increase the fibre content.

Cereal brans are rich in bioactive compounds like antioxidants and phytochemicals that could be extracted for application as an ingredient in nutraceuticals or as functional foods. They can also serve as substrates for microbial fermentation to produce bio-based products such as enzymes, vitamins, organic acids, and other valuable compounds [107,108]. This technology reduces waste and creates value from potential by-products that would otherwise be discarded.

Fermented cereal bran is nutritious and can be recycled as a high-quality animal feed. The fermentation process enhances the digestibility of bran while increasing its protein content, thereby making the fermented bran a good feed for livestock and poultry and reducing dependence on synthetic feed additives [96,109].

The residual cereal bran can be channelled into anaerobic digesters for biofuel production such as bioethanol, biogas, biochar, bio-oil and syngas [110,111]. The produced biofuel could replace fossil fuels across various facilities, thereby minimising their use and increasing the sustainability of the entire process [112,113].

The cereal bran from *Ogi* production can be composted into bio-fertilisers [114,115]. These can be applied to the soil to improve agricultural productivity, thereby increasing soil health and biodiversity, supporting sustainable farming, and reducing the use of chemical fertilisers.

A value addition in this direction is the production of biodegradable packaging materials from the bran and starch by-products of *Ogi* production. This would replace conventional plastic packaging, thereby reducing plastic waste that pollutes the environment. This would provide a full-circle approach whereby waste generated by production is transformed into useful packaging products. Alahmed and Simsek [116] used arabinoxylan extracted from wet-milling corn bran products to produce biodegradable food packaging materials.

#### 6.1.2. Spent Water from Soaking and Milling

Since the wastewater generated during *ogi* production contains high nutrient levels, it can be used to irrigate farmlands or for aquaculture, thereby providing an environmental waste management system and contributing to agricultural productivity.

The aqueous steep liquor is concentrated in soluble proteins (40–50%), amino acids, minerals, and sugars, making it a low-cost nitrogen and carbon source for industrial fermentation media, an established practice in antibiotic, enzyme, and organic acid production [117]. Redirecting this stream from effluent to industrial input would simultaneously reduce wastewater pollution and generate economic value.

### 6.2. Garri: Circular Bioeconomy Integration

*Garri* represents the larger of the two economies by far. Nigerian *garri* sales were estimated at approximately 6 million tonnes in 2011, equivalent to roughly 26 million tonnes of fresh cassava root, about 68% of the country’s total annual cassava production, making it the dominant outlet for Nigeria’s cassava harvest [118]. Production remains overwhelmingly informal and women-led: a recent survey of processors found the industry to be 97.9% female-dominated, generating a gross margin of approximately 28% and return on investment of approximately 39% per processing cycle, despite most processors lacking access to formal credit [119].

*Garri* processing from fresh cassava generates cassava peels (15–20% of tuber weight), starch residues and cassava bagasse, and processing effluents [120]. Nigeria, as the world’s largest cassava producer, generates an estimated 20 million tonnes or more of cassava processing residues annually [121]. Valorisation pathways for *garri* by-products are summarised in Table 2.

#### 6.2.1. Peels from the Initial Processing of Cassava Tubers

Although the use of energy in various sectors, industrial, agricultural, domestic, transportation, and other commercial, has increased, the reliance on fossil fuels as the main energy source has led to the emergence of global issues like environmental degradation and global warming [122,129]. The most common biofuel utilised in the transportation industry is bioethanol. The bioethanol production by Sokan-Adeaga et al. [122] revealed that cassava peels could produce reasonable ethanol. The cassava-to-ethanol conversion process has been well established in China, Thailand, and West Africa [120]. Efeovbokhan et al. [130] also reported the production of bioethanol and biogas using cassava peels; thus, sustainable energy is becoming more utilised. Bioethanol and biogas production from cassava peels similarly contributes to SDG 7 by diversifying rural and peri-urban energy supply away from fossil fuels, with the added climate co-benefit of displacing open burning or uncontrolled decomposition of peel waste, both of which generate uncontrolled methane and CO_2_ emissions.

Most of the wood used in the paper business comes from natural forests, but many pulp companies fail to plant new trees [126]. The cassava peel is rich in cellulose, one of the three primary components of raw materials for pulp: cellulose, hemicellulose, and lignin. Cassava by-products are cheap, easily accessible, abundant, low-density, renewable, sustainable, and biocompatible. According to Kurnia et al. [126], the three analyses of paper made from cassava peel pulp showed that cassava peel can be used as a mixed ingredient in papermaking.

Cassava peels, particularly the bitter cassava varieties, contain high levels of Hydrogen cyanide (HCN) and are poisonous if not properly processed. Sun drying is an effective way to significantly reduce HCN levels. In rural Nigeria, peels are often given away to workers who peel cassava roots, so the peels can be dried, fed to their animals, especially goats, and used for fishmeal processing [101]. Cassava peels can make livestock production cheaper, safer, more consistent, and more easily accessible. It also reduces the menace of environmental degradation.

The application of biochar to soil is a sustainable approach to enhance soil quality and fertility. Sustainable management of large volumes of agricultural and industrial waste is possible through their valorisation as biochar. Cassava peels obtained during *garri* production are considered viable agro-waste for energy recovery [124]. It has been reported that the biochar mineral content of cassava peel waste is acceptable, with a carbon content of 48.7 wt% and significant levels of sodium, calcium, potassium, and nitrogen [125].

The development of bioplastics from cassava offers a bio-based alternative to plastics, which may reduce carbon emissions. Moreover, the waste management of bioplastics differs from that of petroleum-based plastics [128].

#### 6.2.2. Settling Starch Residues and Cassava Processing Bagasse

Some starch present in the squeezed water after fermenting cassava for garri production is collected, allowed to settle, and recovered by pooling out the sediment. The settled starch is edible in some regions of Nigeria and is also useful in the textile industry and fabric laundering. The cassava bagasse, a typical solid residue of cassava processing, contains between 40.1% and 75.1% starch (dry weight) and between 14.9% and 50.6% fiber [120].

#### 6.2.3. Wastewater Effluents

To reduce water usage, the cleaning water generated during *garri* production is often recycled and reused on the production line. Adekunle et al. [127] reported that one ton of cassava wastewater can yield 28 m^3^ of biomethane. Recovering biomethane from *garri* processing wastewater, rather than discharging it untreated, directly supports SDG 6 (Clean Water and Sanitation), which targets improved water quality through reduced pollution and the halving of untreated wastewater discharge, and SDG 7 (Affordable and Clean Energy), through the substitution of biomethane for fossil-derived energy sources in processing communities.

Cassava wastes and peels can also serve as substrates for producing organic acids such as citric acid, lactic acid, succinic acid, volatile fatty acids, and acetic acid through fermentation [123]. These products are beneficial, particularly in the pharmaceutical, chemical, and food industries. Zhang et al. [120] confirmed that cassava industrial wastes produce various organic compounds. Cassava peels are rich in carbohydrates, including starch, cellulose, hemicellulose, and pectin, which can serve as a carbon source for microbial growth and enzyme production [131].

The pathways described above are technically demonstrated, largely at laboratory or pilot scale; their translation into functioning value chains faces three practical constraints not yet addressed in the literature reviewed here. First, *ogi* and *garri* production in Nigeria is overwhelmingly artisanal and geographically dispersed across small household- and community-scale processors, so by-product streams such as *ogi* bran and steep liquor arise in small, scattered volumes rather than the concentrated streams assumed by most valorisation studies; collection, aggregation, and transport logistics, rather than the underlying conversion technology, are likely to be the binding constraint on commercial-scale recovery. Second, energy balance is a genuine concern for some routes: bioethanol production from cassava peels requires drying, hydrolysis, and distillation steps that are themselves energy-intensive, and net energy gain depends heavily on feedstock moisture content and process design; without techno-economic and life-cycle assessment specific to small-scale Nigerian operating conditions, it cannot be assumed that every proposed pathway is net energy-positive at artisanal scale. Third, Nigeria currently lacks dedicated regulatory or extension infrastructure for agro-industrial by-product valorisation: there is no equivalent of an established biomass collection network, and NAFDAC and relevant state agencies have not yet issued specific standards or incentive schemes for by-product-derived food, feed, or biomaterial products, which limits investment certainty for potential processors. Addressing these constraints will require aggregation-point infrastructure at existing processing clusters, context-specific techno-economic and energy-balance studies rather than extrapolation from industrial-scale data, and policy attention alongside the technical research priorities.

## 7. Challenges, Constraints, and Critical Gaps

### 7.1. Food Safety and Microbial Hazards

Food safety is the most immediate challenge facing Nigerian fermented foods in both domestic and international markets. Reliance on spontaneous fermentation with undefined microbial communities creates batch-to-batch variability in quality and safety. Mycotoxin contamination, particularly aflatoxins in maize-based products such as *ogi*, and ochratoxin A in sorghum-based beverages, is well-documented and public health-relevant [32,42]. Aflatoxin levels exceeding regulatory limits have been reported in *ogi* and grain-based beverages sampled from Nigerian markets, highlighting the need for integrated pre- and post-harvest management.

The absence of effective national regulatory enforcement of traditional fermented foods compounds these risks. Unlike industrial food products subject to NAFDAC oversight, the vast majority of traditionally produced fermented foods enter commerce through informal channels and undergo no systematic safety testing.

### 7.2. Standardisation and Process Control

Fermentation processes for Nigerian traditional foods vary substantially in duration, temperature, substrate preparation, and microbial inoculum, affecting quality, safety, and nutritional composition. This variability, acceptable within community settings, becomes a major barrier to commercialisation. Development of defined starter cultures for *ogi*, *garri*, and *iru* is technically feasible and has been demonstrated at the research scale [3], but translation to artisanal and SME-level production requires investment in training, cold-chain infrastructure for starter-culture distribution, and economic incentives for adoption.

### 7.3. Industrialisation, Regulatory, and Intellectual Property Frameworks

A dimension almost entirely absent from the existing literature is the protection of intellectual property and traditional knowledge. As Nigerian fermented foods attract growing scientific and commercial interest, questions of who benefits from commercialisation become critically important. Geographical indications (GIs) could provide a framework for protecting the cultural and economic value of distinctively Nigerian products, while regulatory frameworks specifically adapted to traditional fermented foods, acknowledging regional and cultural variability without imposing inappropriately onerous burdens on small-scale producers, are urgently needed [132].

### 7.4. Research Priorities

Three priority research directions are identified based on this review.

Application of metagenomics and metabolomics to Nigerian fermented foods is still in its early stages. Comprehensive profiling of the microbial ecology and metabolic activity of key products is essential for understanding determinants of quality, safety, and nutritional value, and for developing effective starter cultures. This is positioned as upstream, enabling work; neither food safety surveillance nor standardisation can be designed rationally while the microbial communities responsible for fermentation, and the hazards specific to each product and process, remain incompletely characterised.

Defined starter cultures tailored to the processing conditions and sensory expectations of Nigerian consumers need to be developed and validated for safety, stability, and performance under the variable temperature and humidity conditions of tropical processing environments.

The economic contribution of fermented food production to rural livelihoods, particularly women’s livelihoods, remains poorly quantified at scale. Household-level studies integrating nutritional, economic, and gender dimensions are needed to inform targeted policy support. Any intervention on safety, standardisation, or bioeconomy integration that is designed without accounting for gender dynamics in production risks being either ineffective or, in constraining women’s already limited economic returns further, actively harmful to their livelihoods.

## 8. Conclusions and Future Perspectives

This review has demonstrated that the ethnogeographic diversity of Nigerian fermented foods reflects the coherent interplay of ecology, agriculture, ethnicity, and history across the diverse regions in Nigeria. Understanding these drivers is a prerequisite for designing contextually appropriate strategies for food safety improvement, process standardisation, and commercialisation that respect the traditional knowledge systems that underpin these foods.

The circular bioeconomy framework provides a compelling lens for repositioning Nigerian fermented foods within global sustainability discourse. *Garri* and *ogi* exemplify how traditional food systems can serve as prototypes for resource-efficient, low-waste production models that generate multiple value streams from a single agricultural commodity. Realising this potential requires coordinated investment in infrastructure, research, regulatory capacity, and gender-equitable value-chain development.

Metagenomics-based microbiome characterisation, systematic nutritional assessment under controlled conditions, techno-economic analysis of bioeconomy integration at relevant scales, and development of appropriate regulatory frameworks are all identified as priority areas. International collaboration between Nigerian research institutions and partners with advanced analytical capacity will be essential. Nigerian fermented food traditions, if given the research, policy, and commercial attention they merit, represent not only a cultural heritage worthy of preservation but a genuine asset for sustainable food system transformation in Sub-Saharan Africa and beyond.

## Figures and Tables

**Table 1:** Nigerian fermented foods: substrates, fermentation types, and principal associated microorganisms.

Fermented Foods	Substrates	Forms/ Uses	Principal Microorganisms	Selected References
*Garri*, *Fufu*, *Abacha* and *Lafun*	Cassava	Solid	*Limosilactobacillus fermentum*, *Lactoplantibacillus plantarum*, *Weissella confusa*, *Saccharomyces cerevisiae*, *Pichia scutulata*, *Kluyveromyces marxianus*, *Hansenia guilliermondii*, *Leuconostoc fallax*	[12,13,14,15,16]
*Ogi* and *koko*	Maize, millet, and sorghum	Slurry, gruel	*L. plantarum*, *L. fermentum*, *Lactobacillus cellobiosus*, *Lentilactobacillus buchneri*, *Pediococcus acidilactici*, *P. pentosaceus*, *S. cerevisiae*, *Rhodotorula graminis*, *Candida krusei*, *Candida tropicalis*, *Geotrichum candidum*, *Geotrichum fermentum*	[17,18,19,20,21,22,23]
*Burukutu*, *Pito*	Maize, millet and sorghum	Liquid	*Acetobacter* spp., *Candida* spp., *Enterobacter* spp., *Lactobacillus* spp., *Saccharomyces cerevisiae*, *Saccharomyces chevelieri*, *Leuconostoc mesenteroides*	[24,25,26,27,28,29]
*Kunnu-zaki*	Millet, sorghum	Liquid, breakfast, beverage	*L. fermentum*, *Pediococcus pentosaceus*, *Weisella confusa*, *Enterococcus faecalis*, *Lactobacillus delbrueckii*, *L. plantarum*, *Lacticaseibacillus casei*, *Corynebacterium*, *Lactococcus lactis*, *Saccharomyces cerevisiae*	[30,31,32,33,34,35,36]
*Fura*	Millet	Liquid, beverage, breakfast	*L. fermentum*, *W. confuse*, *Issatchenkia orientalis*, *Saccharomyces cerevisiae*, *Pichia anomala*, *Candida tropicalis*, *Saccharomyces pastorianus*, *Yarrowia lipolytica*, *Galactomyces geotrichum*	[37,38]
*Iru*, *Dawadawa*, *Ogiri* and *Ugba*	Locust bean	Solid	*Bacillus megaterium*, *Bacillus circulans*, *Bacillus licheniformis*, *Bacillus subtilis*, *Bacillus coagulans*, *Leuconostoc* spp., *Staphylococcus* spp.	[3,39,40,41,42,43,44]
*Wara*	Milk	Solid	*Lactobacillus* spp., *Lactococcus* spp., *Leuconostoc* spp., *Pediococcus* spp.	[3,45]
*Agadagidi*	Banana/plantain	Liquid	*Bacillus subtilis*, *Bacillus cereus*, *Lactobacillus plantarum*, *Streptococcus faecalis* and *Leuconostoc mesenteriodes*, *Saccharomyces cerevisiae* and *Candida utilis*	[46]
*Otika*	Sorghum	Liquid, alcoholic beverage	*B. cereus*, *B. subtilis*, *C. krusei*, *C. tropicalis*, *Enterobacter cloacae*, *L. brevis*, *L. fermentum*, *L. plantarum*, *Leuconostoc mesenteroides*, *S. cerevisiae*	[47]
*Emu* (Palm wine)	Oil palm or Raphia palm	Liquid, alcoholic beverage	*Saccharomyces cerevisiae*, *Schizosaccharomyces pombe*, *Geotrichum lactis*, *Debaryomyces hansenii*, *Zygosaccharomyces rouxii*, *Saccharomyces cerevisiae*, *Hanseniaspora uvarum*, *L. plantarum*, *Leuconostoc mesenteroides*, and *L. mesenteroides* subsp. *dextranicum*	[12,48,49,50,51,52]

**Table 2:** Valorisation pathways for *garri* processing by-products within a circular bioeconomy framework.

By-Product Stream	Valorisation Pathway	Value-Added Output	Selected References
Cassava peels	Microbial fermentation/hydrolysis	Bioethanol, biogas, organic acids (citric, lactic, acetic)	[122,123]
Cassava peels	Pyrolysis/thermal treatment	Biochar for soil enrichment (Carbon content ~48.7 wt%)	[124,125]
Cassava peels	Sun-drying/detoxification	Animal feed (goats, fish)	[101]
Cassava peels (cellulose-rich)	Pulp processing	Paper and packaging materials	[126]
Bagasse/starch residue	Starch recovery	Edible starch; textile sizing	[120]
Processing effluent/wastewater	Anaerobic digestion	Biomethane (~28 m^3^ per tonne cassava waste)	[127]
Peels/starch by-products	Bioplastic synthesis	Biodegradable packaging materials	[128]

## Data Availability

No new data were created or analyzed in this study. Data sharing is not applicable to this article.
